# Risk map and spatial determinants of pancreas disease in the marine phase of Norwegian Atlantic salmon farming sites

**DOI:** 10.1186/1746-6148-8-172

**Published:** 2012-09-24

**Authors:** Saraya Tavornpanich, Mathilde Paul, Hildegunn Viljugrein, David Abrial, Daniel Jimenez, Edgar Brun

**Affiliations:** 1 Section for Epidemiology, Department of Health Surveillance, Norwegian Veterinary Institute, Ullevålsveien 68, Pb 750 Sentrum, N-0106, Oslo, Norway; 2 UR AGIRs, Centre de coopération internationale en recherche agronomique pour le développement (CIRAD), TA C22/E, Campus international de Baillarguet, 34398, Montpellier cedex 5, France; 3 Centre for Ecological and Evolutionary Synthesis (CEES), Department of Biology, University of Oslo, P.O. Box 1066, Blindern, N-0316, Oslo, Norway; 4 UR 346, Institut National de la Recherche Agronomique (INRA), 63122, Saint-Genès-Champanelle, France

**Keywords:** Pancreas disease, Aquatic epidemiology, Spatial analysis, Disease mapping, Bayesian modeling

## Abstract

**Background:**

Outbreaks of pancreas disease (PD) greatly contribute to economic losses due to high mortality, control measures, interrupted production cycles, reduced feed conversion and flesh quality in the aquaculture industries in European salmon-producing countries. The overall objective of this study was to evaluate an effect of potential factors contributing to PD occurrence accounting for spatial congruity of neighboring infected sites, and then create quantitative risk maps for predicting PD occurrence. The study population included active Atlantic salmon farming sites located in the coastal area of 6 southern counties of Norway (where most of PD outbreaks have been reported so far) from 1 January 2009 to 31 December 2010.

**Results:**

Using a Bayesian modeling approach, with and without spatial component, the final model included site latitude, site density, PD history, and local biomass density. Clearly, the PD infected sites were spatially clustered; however, the cluster was well explained by the covariates of the final model. Based on the final model, we produced a map presenting the predicted probability of the PD occurrence in the southern part of Norway. Subsequently, the predictive capacity of the final model was validated by comparing the predicted probabilities with the observed PD outbreaks in 2011.

**Conclusions:**

The framework of the study could be applied for spatial studies of other infectious aquatic animal diseases.

## Background

Pancreas disease (PD) is a viral disease affecting Atlantic salmon (*Salmon salar L.*,) and rainbow trout (*Oncorhynchus mykiss*). A necessary cause of the disease is *salmon pancreas disease virus*, an alphavirus often referred as *salmonid alphavirus* (SAV). The onset of a PD outbreak is favored by unspecified influential environmental factors. Diagnostic criteria for PD outbreak were described by Taksdal et al., 2007 [1]. So far, 6 subtypes of SAV have been reported worldwide, whereas, only subtypes 2 and 3 have been detected in Norwegian salmonid farming sites [2, Hilde Sindre per communications]. The disease has been a significant problem in salmonid farming industries in Scotland, Ireland, and Norway [2]. An economic modeling study estimated that the direct cost of PD accounting for biological losses, disease controls and treatments, and insurance payment in a Norwegian salmonid site with an approximate of 500,000 smolts to be at least 14.4 million NOK in 2007 [3].

**Figure 1:**
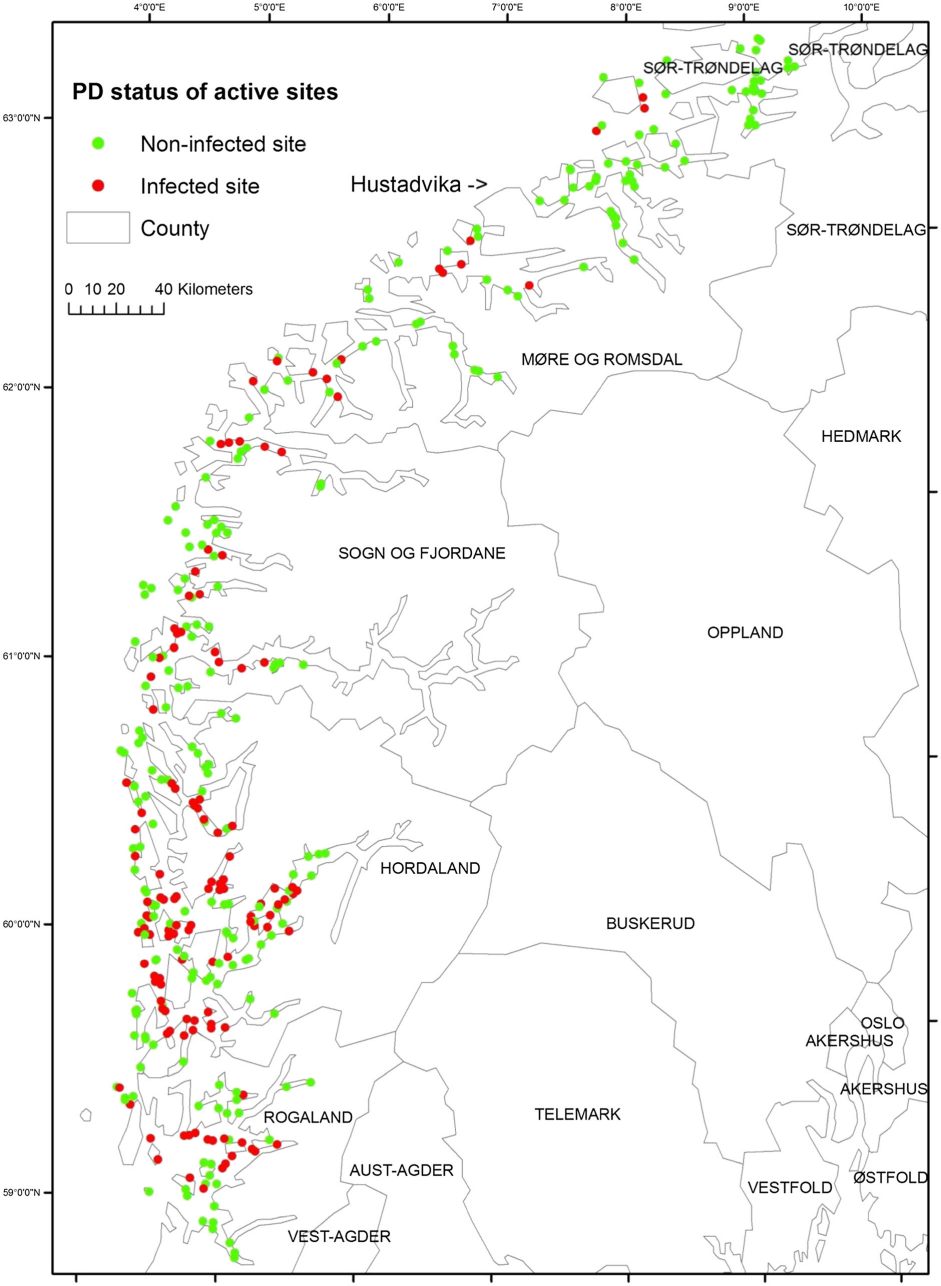
Geographical distribution of 359 active Atlantic salmon farming sites located near 6 counties of Norway (Vest-Agder, Rogaland, Hordaland, Sogn & Fjordane, Møre & Romsdal, SørTrøndelag) from 1 January 2009 to 31 December 2010.

The characteristics of SAV and PD outbreaks have been thoroughly reviewed [4-11]. In Norway, PD outbreaks have been reported only during the marine phase. The disease is regarded as endemic in the area along the western coast of Norway from Rogaland county to a firewall at Hustadvika (Figure 1) in Møre and Romsal with few sporadic outbreaks occurring north of this border. This firewall is a 10 nautical mile exposed costal area without any aquaculture activity. Veterinary authorities together with salmon industries have launched different prevention and control strategies to control the situation in the south and to keep the northern area free of PD [12]. All findings of epidemiological studies of PD outbreaks support the hypothesis that the virus can spread through the water media, causing a PD outbreak both with and without human intervention. This aspect of the disease spreading via water media is a crucial element of aquatic infectious diseases [9,13]. Therefore, it is worthwhile to evaluate the spatial clustering of infected sites in epidemiological studies of PD outbreaks. In this study, we initially evaluated potential risk factors of PD in marine phase of Atlantic salmon farming sites, accounting for spatial congruity of infected sites. Then, the predicted probabilities of PD occurrence based on the model results were displayed in an interpolated map covering the southern part of Norwegian marine aquaculture.

## Methods

### Data

In this study, we included Atlantic salmon farming sites located in 6 Norwegian counties (Vest-Agder, Rogaland, Hordaland, Sogn & Fjordane, Møre & Romsdal, SørTrøndelag), where most of the PD outbreaks were reported so far. To be considered as a PD at-risk site, the included sites were required to rear fish on the site for at least 6 consecutive months during the study period (1 January 2009 to 31 December 2010). All operators of Norwegian salmonid farming industries are required to register production statistical data to responsible authorities on a monthly basis. These statistics are linked to a farm site identity, which is geo-referenced in the Directorate of Fisheries (www. fiskeridir.no). According to the inclusion criteria, 359 Atlantic salmon farming sites were qualified for model estimation of this study. The geographical distribution of the study sites is presented in Figure 1. In addition, a second dataset of active farming sites during a period of 2011 was created for validating the predictive capacity of the model. The second dataset contained 418 farming sites located throughout the study area.

#### PD infection status

All farming sites were monitored by the farmer for unusual increases in fish mortality and activities (e.g. runt, moribund). If PD was suspected, samples were routinely sent for laboratory confirmation using histopathological examination and PCR testing. The study sites were classified as PD positive if at least 1 PD outbreak were confirmed during the study period, and PD negative, if otherwise.

#### Site latitude

In the present study, we evaluated if the site location, based on the latitude, had an influence on the PD occurrence. In this study, the latitudes of the study sites cover the area of 63°39'5,481"N (the farthest north) to 58°12'33,449"N (the farthest south). The value of site latitude was converted to meters prior to analyses.

#### Site density

Site density for a given site was the number of farming sites (excluding the given site) that located within 10-km seaway distance of the given site. In this study, the site density ranged from 1 to14, had mean of 4, and a median of 4.5.

#### Smolt cohort

A smolt cohort is the group of smolts that were put to sea and reared in sea cages until slaughtering. We identified the time when the smolt cohorts were put to sea. If the cohort was put to sea in the period of August-February it was classified as ‘a cohort of autumn smolt’; otherwise, if the cohort was put to sea in the period of March-July, it was classified as ‘a cohort of spring smolt’. Of the 359 study sites, 168 (46.8%) sites had only autumn cohorts; 170 (47.4%) sites had only spring cohorts; and 21 (5.8%) sites had mixed cohorts (both cohorts of autumn and spring smolts).

#### PD history in 2008

Previous recent history of PD in a nearby area is likely to have an impact on a future probability of PD occurrence. In this study, we evaluated this effect by classifying the study sites based on whether or not they were located within 10-km seaway distance of sites where PD was suspected or confirmed in 2008. The 10-km seaway distance was used as the cutoff because it was estimated that sites locating within 10-km seaway distance of PD infected sites had a large impact on PD risk [14]. Of 359 study sites, 225 (63%) sites were located within 10-km radius of PD suspected/confirmed sites in 2008. Furthermore, among these 225 sites, 110 (49%) sites were classified as PD confirmed cases during the study period.

#### Local biomass density

The local biomass density (LBD) indicates the potential of infection endangerment at a given site. This factor depends on average biomass of the surrounding sites to a given site (excluding LBD of the given site). The LBD data were estimated for all registered salmon fish farming sites in Norway and the estimation method was described by Jansen et al (2012) [15]. In the present study, we evaluated the effect of LBD on the probability of PD occurrence at each farming site. The LBD of the study sites ranged from 4.87e + 02 to 4.62e + 08; had a mean of 2.19e + 08; and median of 2.11e + 08. For the analysis purpose (facilitating convergence of the model), the LBD was transformed using log10, and then back transformed for interpretation.

### Statistical analysis

#### Univariate and multivariate analysis

All regression analyses were performed using WinBUGS (windows version of Bayesian inference using Gibbs Sampling) open source software [16]. First, we performed univariate logistic regression to individually evaluate the effect of each potential risk factor on PD occurrence. Records of PD confirmed cases from 1 January 2009 to 31 December 2010 were used as a dependent variable of the model. The evaluated risk factors were site latitude, site density within 10-km seaway distance, smolt cohort, PD history in 2008, and LBD. We explored the assumption of linear relationships with generalized additive models, using the mgcv package in R software. Variables with a 80% posterior probability interval including zero were excluded from further analyses. Then, multivariate logistic regression models were performed with and without a spatial component. A stepwise selection based on the Deviance Information Criterion (DIC) was used for selecting the combination of the covariates; the model with lower DIC is preferred to the model with larger DIC [17]. DIC was also used for evaluating whether adding spatial random effect would improve model fit in the final model. Residuals of all models were in turn checked for spatial dependency, as any pattern present in the residuals of a statistical model may indicate that results are biased [18]. To do so, we applied a Monte Carlo method [19,20] using the geoRglm package of the R software. This consists in comparing the observed variogram with variogram ‘envelopes’ that were computed by simulating 999 permutations of the data values across locations.

#### Spatial dependency modeling

We grouped fish farming sites into hexagonal subunits which were included in the model as a spatial area-level random effect [21]. Nineteen hexagons (width of 80 km, corresponding to 5546.6 km^2^) were created; they contained between 1 and 52 farming sites (mean = 35). The area-level effect was modeled using a Conditional Autoregressive (CAR) prior structure, in which an adjacency matrix was specified with a weight of 1 given to adjacent hexagons and a weight of 0 given to nonadjacent hexagons.

Briefly, each site in this study was labeled with i = 1…19 (the hexagon in which the site is localised), and j = 1…359 (the unique site identifier). The observed case of PD outbreaks at each site (y_[ij]_) during the study period followed a Bernoulli distribution. The model can be written as the following equations:

(1) $${\text{y}}_{\left[\text{ij}\right]}\sim \text{Bernoulli}\left(\pi_{\left[\text{ij}\right]}\right)$$

(2) $$\text{logit}\left(\pi_{\left[\text{ij}\right]}\right))=\alpha +\beta {\text{x}}_{\left[\text{ij}\right]}+{\text{v}}_{\left[\text{i}\right]}$$

(3) $${\text{v}}_{\left[\text{i}\right]}\sim \text{Normal}\left(\mu_{\delta \left[\text{i}\right]},1/\sigma^{2}\right)$$

*β* is the vector of coefficients, x_[ij]_ is the covariates matrix observed on site j, *v*_[i]_ is the random area-level effect which is spatially structured according to the model of Besag et al (1991) [22]. The mean of this model is driven by the average risk level of the adjacent hexagon set δ_[i]_ of the hexagon i. The precision (σ^2^) of *v*_[i]_ follow a gamma distribution. Priors for model coefficients were based on a normal distribution (with a mean of 0.5 and a precision of 5e-04). We implemented the models in WinBUGS, and estimated the model parameters using Gibbs sampling, which is the method for simulating a new value for the model parameters from its full posterior conditional distribution, given the current values for the remaining parameters [23]. In this study, the model parameters were estimated based on 45000 effective iterations. An additional first 5000 iterations were discarded as a burn-in period. Geweke and Heidelberger-Welch tests were used to assess the convergence of the models [16].

### Interpolated map of PD predicted probability

The median value (50^th^) of the posterior distribution of the probability estimate of PD occurrence, obtained from the final Bayesian logistic model, was used for creating a PD risk map. First, the empirical semi-variogram of the point estimates was plotted. Several statistical models (exponential, Gaussian, spherical) were investigated to identify the best-fit variogram model. Those parameters were then used to interpolate values between all the 359 farming sites considering a fixed radius of 40 km and a cell size of 500 m. An ordinary spatial kriging was used and allowed to produce a raster map; the variance of output raster was also mapped. Kriging is a group of geostatistical techniques for interpolating the value of a random field at an unobserved location based on observed values at nearby locations; it has been widely used in veterinary epidemiology [20]. Kriging and mapping were performed with the gstat package of the R software and the spatial analyst extension of ArcGIS software v.9.3 (ESRI Inc. Redlands, CA USA).

### Sensitivity analysis

The threshold of 80 km, which was used to define the width of the spatial grid, was selected in order to have an acceptable number of farming sites per hexagon. To see whether the size of the grid would influence the model outputs, we carried out a sensitivity analysis by running the final model with a grid size of 40 km. As this size produced hexagons without any farming sites, the empty adjacent hexagons were merged. This resulted in a final grid of 39 polygons, which were used in the model. DIC and effect estimates of this model were compared to those of the model of originally selected grid size.

### Model validation

Relative operation characteristic (ROC) statistical methodology was used to validate the accuracy of the model. The approach has been widely used to access performance of diagnostic tests of animal diseases, and has been applied for validation of models predicting spatial distributions [24-27]. For the present study, the ROC curve presents the plot of the “sensitivity” (true-positive value) versus “specificity” (true-negative value) at each plotted point. The sensitivity is the probability that the model predicts that a site is infected when that site is truly infected. The specificity is the probability that the model predicts that a site is not infected when that site is truly not infected. Each plotted point reflects the average probability that infection will be present on a site. We plotted ROC and calculated an area under the curve (AUC) to check the model fit by comparing the estimated probability of PD occurrence and the observed PD status during the study period. Also, we compared the predicted probabilities based on the final model with the observed PD status in 2011 in order to evaluate if this model could be used for predicting future outbreaks. If the PD occurrence is 100% predicted by the model, AUC of 1 is obtained [24]. All those performance measures were calculated using the pROC package of the R software [28].

## Results

### Univariate and multivariate analyses

A total of 359 salmon fish farming sites were included in the present study based on the inclusion criteria. Of this study population, 127 sites (35%) were classified as PD cases during the study period. Geographical distribution of the study sites classified by PD status is presented in Figure 1. DIC and coefficient estimates from the univariate logistic regression analysis are presented in Table 1. According to our significance criteria, all covariates except the smolt cohort were further evaluated in multivariate analysis. Table 2 presents the model covariates and DIC of various models depending on combinations of covariates.

**Table 1:** Univariate analysis using a Bayesian approach of potential risk factors for PD occurrence of 359 Atlantic salmon farming sites located in the southern part of Norway from 2009 to 2010

**Model covariates**	**Median (80%PI)**	**DIC**
intercept	-0.60 (-0.75, -0.46)	468.51
intercept + spatial component	NA	440.03
intercept + latitude	-0.62 (-0.80, -0.45)	442.67
intercept + site density	0.30 (0.19, 0.42)	459.14
intercept + smolt cohort	0.13 (-0.15, 0.41)	469.99
intercept + PD history	1.89 (1.53, 2.29)	417.79
intercept + LBD	2.40 (1.85, 3.02)	440.20

*NA* = Not available, *PI* = Probability interval.

**Table 2:** Multivariate analysis using a Bayesian approach of potential risk factors for PD occurrence of 359 Atlantic salmon farming sites located in the southern part of Norway from 2009 to 2010

**Model covariates**	**DIC**
intercept + latitude + site density + PD history + LBD + spatial component	402.79
intercept + latitude + site density + PD history + LBD ***	401.97
intercept + site density + PD history + LBD	408.05
intercept + latitude + PD history + LBD	403.19
intercept + PD history + LBD	410.71
intercept + LBD + spatial component	414.99
intercept + PD history + LBD + spatial component	409.78
intercept + site density + PD history + LBD + spatial component	408.36

*** final model.

There was no evidence of multicollinearity among the covariates. Linear correlation between LBD and site density was moderate (Pearson coefficient equals to 0.63 (*p* < 0.05). A generalized additive model of LBD as a nonlinear function of site density showed a linear positive association for small site densities, the association leveling off for the higher site densities. The final model consisted of site latitude, site density, PD history, and LBD. Although there was an evidence of a spatial cluster of PD infected sites, which was indicated by a decrease of DIC after adding a spatial component to a model with an intercept alone (Table 1), the PD predicted probability was well explained by the covariates of the final model and the spatial component was finally removed. A plot of empirical semivariogram of model residuals and simulation envelopes (data not shown here) confirmed that the spatial pattern of PD cases was well accounted for by the final model. According to the final model, the site latitude alone had a negative effect on the PD probability (median = -0.42 with 95% probability interval between -0.73 and -0.15) meaning that the risk is increasing as one goes south. Having a history of surrounding sites with PD suspected/confirmed the previous year would increase the risk of getting PD (median = 1.22, 95% probability interval between 0.58 and 1.89). Increasing LBD 10 times of an average LBD would increase the risk of getting PD by 1.24 (95% a probability interval between 0.04 and -2.59). At last, increasing site density by adding one more site in a 10-km seaway distance would slightly increase the risk of getting PD (median = 0.08 with a 95% probability interval between -0.03 and 0.18).

### Probability estimates of PD occurrence

**Figure 2:**
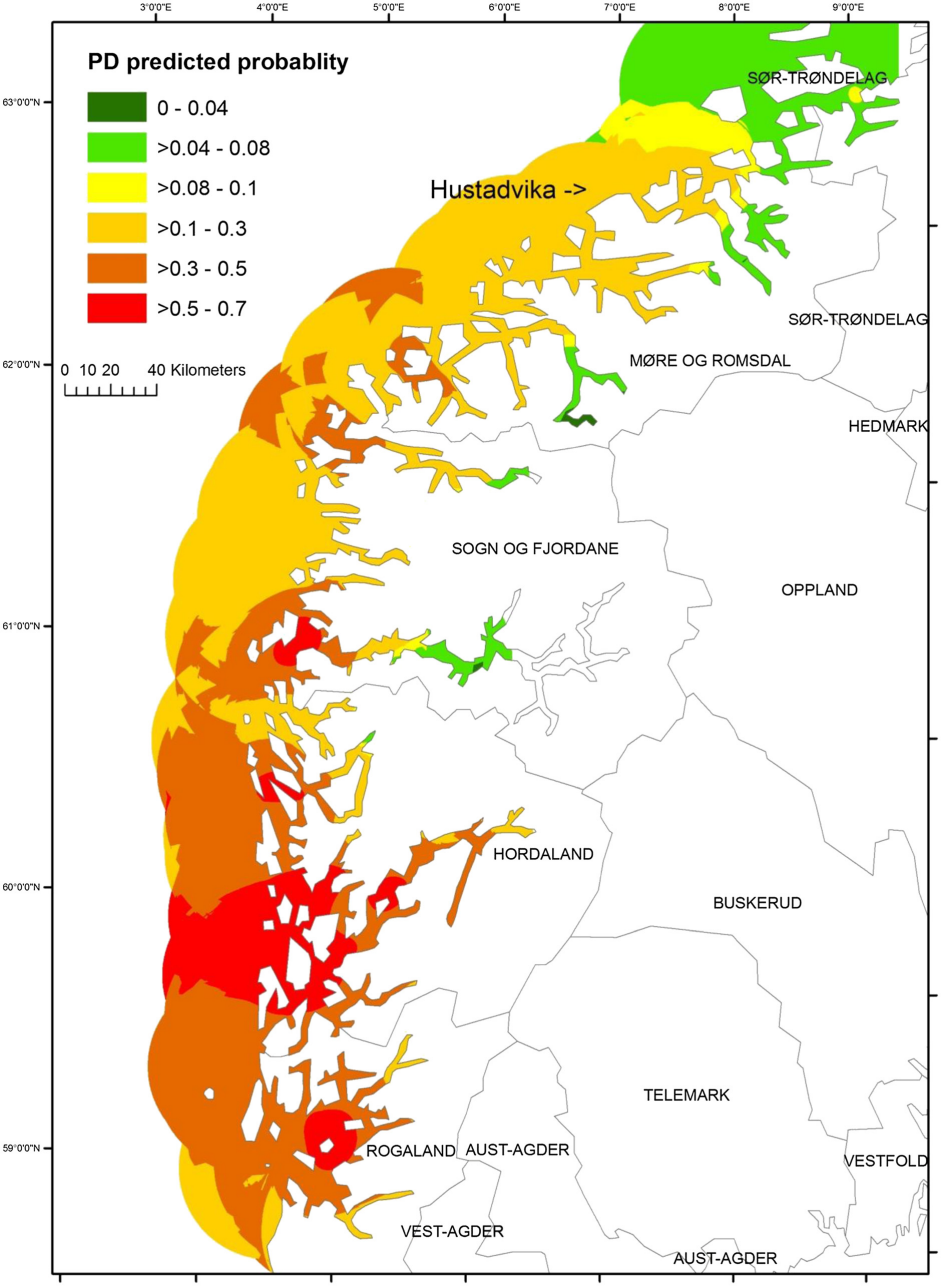
An interpolated map presenting PD predicted probability based on the final model of the present study.

Based on the results of the final model, probability estimates of PD occurrence of the study sites ranged from 0 to 0.75 with a median of 0.4, and about 34% of the study sites had the probability estimates of ≥ 0.5. An interpolated map of the PD predicted probability based on the final model covering the southern part of Norway is presented in Figure 2. To improve the legibility of the map, we classified the predicted probability into 6 categories ranging from 0 to 1.0. The variance of output raster, which helps assessing the uncertainty of the PD predicted probability estimates produced by kriging, ranged from 1-5%. The predictions are more precise in the center of the circles than at their edges (Appendix 1).

### Model validation

**Figure 3:**
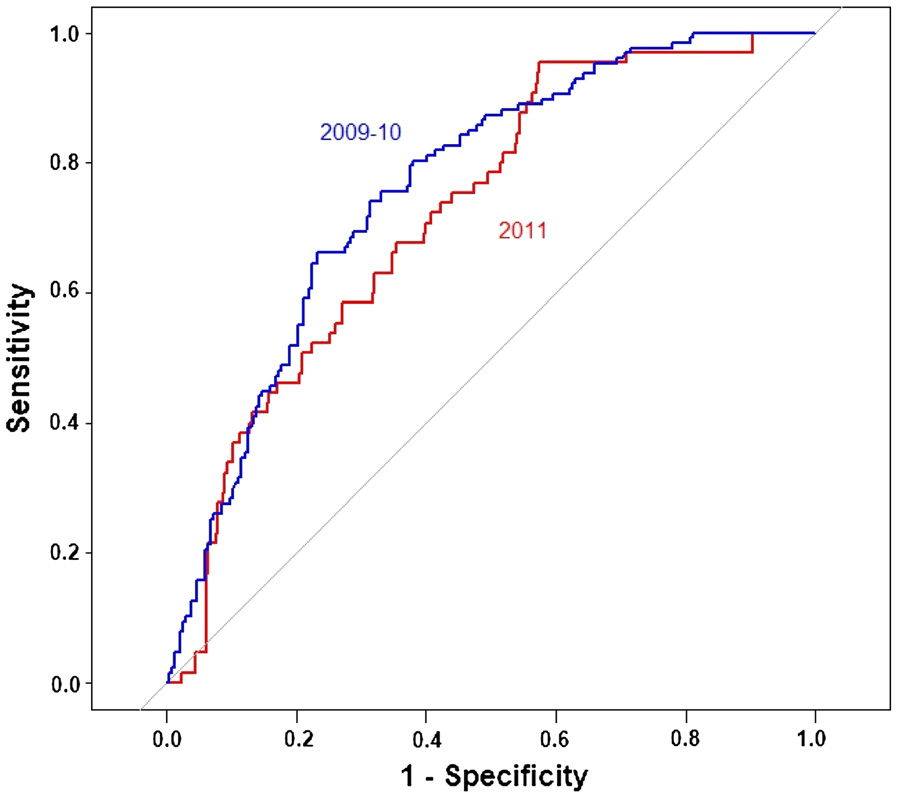
**ROC curves for a) validation of the model estimates with observed PD occurrence in time period 2009-2010 (AUC = 0.76, 95%CI: 0.71-0.81), b) validation of the model prediction with observed PD occurrence in 2011 (AUC = 0.72, 95%CI: 0.66-0.78).** Each plotted point reflects the average probability that infection will be present on a site.

Figure 3 shows the ROC curves for validating the capacity of the final model for: a) model estimation using observed PD data (359 sites) from 2009 to 2010, b) model prediction using observed PD data of 2011 (438 sites). A reasonable predictive capacity of the final model was observed. The AUC were 0.76 (95%CI: 0.71-0.81), and 0.72 (95%CI: 0.66-0.78) for the model estimation, and the model prediction, respectively.

### Sensitivity analysis

For the model with only a spatial component, changing the grid size yielded different estimate of model DICs. The models corresponding to a width of 40 km and 80 km yielded DICs of 424, and 440, respectively. However, changing grid size presented similar DICs and model estimates in the full model (Table 3). This indicates that the effect of spatial congruity on PD occurrence, if occurred, was not influenced by changing of grid size when other significant explanatory variables were included in the model.

**Table 3:** Sensitivity analysis of the effect of changing grid size on DIC and coefficient estimates of the final model

	**40 km grid size (95% PI)**	**80 km grid size (95% PI)**
intercept	-7.74 (-20.61, -1.24)	-9.22 (-19.5, -0.22)
latitude	-0.42 (-0.83, -0.10)	-0.42 (-0.73, -0.12)
site density	0.09 (-0.03, 0.20)	0.08 (-0.03, 0.19)
PD history	1.23 (0.58, 1.90)	1.23 (0.58, 1.89)
LBD	1.00 (0.21, 2.55)	1.19 (0.02, 2.42)
DIC	403.79	402.79

*PI* = Probability interval.

## Discussion

In the present study, we created quantitative PD risk maps of Norwegian Atlantic salmon farming sites accounting for influential factors of PD occurrence at site level. The study was based on the observed data from 1 January 2009 to 31 December 2010. The influential factors included site latitude, site density, PD history, and local biomass density. Subsequently, the probabilities of PD occurrence at site level were displayed in a map covering the southern part of Norwegian marine aquaculture, and validated with observed data of 2011.

The present study included sites located in the southern part of Norway where PD is endemic as well as sites in the southernmost part of the non-endemic area bordering the endemic zone. By including this non-endemic area, the strength of the model to predict the development of PD in this area could be observed. PD strategies in the PD endemic area mainly focus on control and prevention of the disease spreading outside of the affected sites and the endemic area. In the endemic area, with movement restriction, the PD-affected sites have been allowed to keep their fish until slaughtering time. Vaccination has been practiced in the majority of the sites in this PD endemic area; however, the efficacy and effectiveness of the currently used vaccine is debatable [29]. Conversely, vaccination is not a common practice in the PD non-endemic area (the northern part of Norway), but a stamping out policy is regulated and followed by a fallowing period [12]. So far, relatively few cases of PD have been detected in the non-endemic area; therefore, controlling PD outbreak in this area has been manageable financially. Nonetheless, the confining of PD outbreaks within the endemic area may not be feasible in the near future. A great number of financial supports and a strong commitment of fish farming industries would be needed if more stringent control strategies would be applied to prevent the disease spreading northward and to minimize the number of PD outbreaks in the endemic area. An economic model accessing a merit of new disease control strategies would be an assistant tool for decision makers.

The present study showed that the recent history of PD was a useful predictive factor of upcoming PD outbreaks. We found a positive association between the occurrence of PD outbreaks in 2009-2010 and the sites located within 10-km seaway distance of PD suspected/confirmed sites in 2008. This result supports the findings that increasing risk of getting PD is associated with a close distance to infected farming sites [7,14]. We explored the influence of LBD and site density on PD occurrence, and found that these variables also yielded a positive association with increasing risk of getting PD after adjusting for the effects of the other variables. Both variables (LBD, site density) present a concentration of fish; however, they could yield different interpretations. A large site density could have a low LBD if the biomass of neighboring sites is low. The large site density means that there are many sites located in a certain area (in this case 10 km seaway distance). Therefore, frequent visits or multiple activities, such as movement of live fish by a well boat, leading to a higher chance of introducing and spreading the disease agent. On the other hand, a small number of sites each with a large biomass could result in a large overall value of LBD being likely to affect the spreading of virus more than introducing it.

The present study found a non-significant association between the time when smolt were put to sea and the PD occurrence. It has been hypothesized that the cohorts of autumn smolts were more susceptible to PD occurrence than cohorts of spring smolts due to a smaller starting weight of the fish, a lower seawater temperature and a shorter day length, etc [7]. The previous study used a fish cohort as a unit of analysis, and included the cohorts since 2002, which could introduce confounding due to the changes in site management and environment over a period of time. Further works are needed to explore in more details the effect of time when smolt cohorts are put to sea on PD occurrence.

Our results showed a significant effect of latitude, with the risk of getting PD increasing as one goes south. Interpretation of this result is sensitive as latitude can be correlated with several unmeasured variables related to PD risk, including seawater temperature. Exploring the effect of seasonality and seawater temperature on the PD occurrence would be of great interest. However, a cyclical pattern and large missing records of this variable in our study population made its integration into our spatial model challenging. An evaluation of this variable using a subset of the study population was attempted by assessing the proportion of months when the seawater temperature is suitable for survival of SAV [30]. Partly due to a similar pattern of seawater temperature of the subset of the study population we did not find this variable significant (data not shown here). A further evaluation of the effect of this variable on the PD occurrence would require a selection of farming sites with a difference in the pattern of seawater temperature, good-quality and comprehensive data, and would be facilitated by a more dynamic time-scale incorporated into the spatial analysis.

Other research groups have conducted epidemiological studies to determine risk factors of PD transmission [5,31,32]. A close proximity to infected sites, sharing the same water current network, sharing the same equipment with infected sites, sharing of farm ownerships, movement of fish and well boats, inadequate fallowing period, and abundance of sea lice were suggested as potential PD risk factor. This knowledge is invaluable; however, the accessibility and data quality for an extensive area is limited. Another challenging aspect is to clearly distinguish between the risk factors influencing SAV transmission and those inducing PD outbreak.

Peeler and Taylor (2011) published a thorough review emphasizing importance of applying epidemiology tools in a study of aquatic animal diseases, and mentioned a little use of tools describing a spread of infectious disease [33]. In the case of PD, a close proximity of PD-infected sites is clearly a risk factor for the disease transmission [7,14], and sea currents can greatly contribute to spread of the virus between sites [9,13]. Spatial autocorrelation often occurs in outbreaks of infectious diseases in which a farming site located closer to other infected sites is likely to experience an increased chance of acquiring the disease. If this spatial correlation exists and is not accounted for in the model, it could lead to biased parameter estimates and overly optimistic standard errors [20,34]. In our case, despite the spatial clustering of PD infected sites, we did not find a significant improve by adding a spatial component to the final model. This indicates that the predictor variables appropriately accounted for the local spatial correlation.

The use of a Bayesian modeling approach in this work instead of a frequentist approach for estimating the probability of PD occurrence offers a few advantages. It is a flexible tool for accounting for hierarchical levels, such as spatial dependencies [35]. The method allows incorporating previous information providing an appropriate setting for complex models and missing data problem, and the approach yields results in a form of probability distribution that is interpretable intuitively. In our study, non-informative priors were used throughout due to uncertainty of previous information of the parameter estimates. However, the model estimates obtained from this present study could be later used as the prior knowledge to update the future prediction of PD occurrence. The outputs of the Bayesian method gave the posterior distribution of monitoring parameters. We extracted the median of the posterior distribution of the probability of PD occurrence, and displayed in a so-called “PD risk map”. The raster map of PD risk that we produced made it possible to identify local “cold” (green color) and “hot” spots (red color) of PD risk in the area where PD outbreaks area commonly occurring. Based on the PD risk map, the PD predicted probability was high (> 0.5) in the area of Hordaland and some area of Rogaland [Figure 2. In contrast, the probability in Møre and Romsdal was moderate. Without considering the site density and LBD, one could mistakenly believe that there is a higher PD risk in Møre and Romsdal based solely on observed data.

Area under the curve indicated that the raster map produced from data collected in 2009-2010 had a reasonable capacity in discriminating infected farming sites from non-infected ones for the year 2011. This provided a considerable confidence that the risk map could be used as a tool to select areas with an acceptable probability of PD occurrence for salmon farming sites, and to focus surveillance and control measures on high-risk areas. The spatial congruity of infectious aquatic animal diseases is undeveloped and could be explored more in the future by including the use of time-dynamic spatial risk models, and the investigation of the spread of pathogenic agents (with no clinical outbreak) using intensive screening programs. The framework of the study could be applied for spatial studies of other infectious aquatic animal diseases.

## Conclusions

This study presents quantitative risk maps of pancreas disease of Atlantic salmon in Norway. It also provides an insight into the relationship between the risk of PD outbreaks in farming sites and several spatial determinants, including latitude, site density, recent history of PD in a nearby area and local biomass density. The framework of the study could be applied for spatial studies of other infectious aquatic animal diseases.

## Appendix 1

Variance of PD predicted probability.

## Competing interests

The authors declare that they have no competing interests.

## Authors’ contributions

ST coordinated the work, performed all statistical analyses of the present study including ROC/AUC, and drafted the manuscript. MP contributed to development of the Bayesian code, participated in the analyses of the spatial aspect of the study, and assisted in manuscript writing. HV and EB were involved in the study design, analyses of potential risk factors, and assisted in manuscript writing. DA contributed to development of the Bayesian approach. DJ carried out the handling of the raw data, and assisted in performing descriptive statistics of each variable evaluated in the study. All authors read and approved the final manuscript.
